# Clarifying AI-attributable diagnostic gain across separately prepared slides in Techcyte-assisted coproparasitology

**DOI:** 10.1128/jcm.01076-26

**Published:** 2026-10-27

**Authors:** Muh Taufiq, Kevin G. Pascoal

**Affiliations:** 1 Department of Medical Laboratory Technology, Poltekkes Kemenkes Manado 470078, Manado, Indonesia; Mayo Clinic Minnesota, Rochester, Minnesota, USA

**Keywords:** artificial intelligence, coproparasitology, digital microscopy, diagnostic gain, Techcyte

## LETTER

We read with interest the study by Rubio Maturana et al. evaluating the Techcyte artificial intelligence (AI)-assisted system for coproparasitological diagnosis in 178 clinical stool specimens (1). The authors reported additional parasite etiologies in 74 specimens after AI analysis and expert review. We commend this clinically relevant evaluation and its transparent separation of raw algorithmic output from operator-reviewed findings. We suggest, however, that “diagnostic gain” requires more specific attribution when conventional and AI-assisted workflows examine separately prepared slides.

Both workflows used the same concentrated stool pellet, but conventional microscopy and Techcyte analysis evaluated different preparations. Conventional microscopy used a separate drop preparation under a 24 × 60 mm coverslip, whereas the Techcyte slide contained 15 µL of pellet under a 20 × 20 mm coverslip (1). The biological material available to each workflow was therefore not analytically equivalent. Differences in the quantity and processing of stool material can affect diagnostic yield in automated parasite-detection workflows (2). An organism observed only on the Techcyte slide may consequently reflect AI-assisted localization, unequal organism availability between preparations, or both.

The authors appropriately recognized stochastic distribution and low analyzed volume as potential causes of missed detection. In their reproducibility analysis, helminths were detected in only 49 of 78 evaluable replicate slides prepared from eight specimens with very low burdens (1). These findings cannot be generalized to the predominantly protozoan additional detections, but they demonstrate that detectability can change across slides prepared from the same pellet. Importantly, expert review confirms the morphology of an object on the Techcyte preparation; it cannot establish that the same object was present but overlooked on the conventional preparation.

Recent AI-microscopy studies illustrate how attribution can be strengthened. Lundin et al. (3) compared AI with manual microscopy on the same Kato-Katz smears and visually confirmed 79 manual-negative/AI-positive findings within the shared preparation. In a Techcyte wet-mount experiment, Mathison et al. (4) had technologists read each slide before immediately scanning that identical preparation for software analysis. Same-slide comparisons must still control temporal effects: von Bahr et al. (5) found that manual reading before scanning could alter detectability through clearing and hookworm disintegration and recommended delayed, repeated, or randomized reading sequences.

Future Techcyte evaluations could therefore combine same-slide paired assessment, blinded readers, randomized reading order, and replicate-slide allocation using equal volumes. Independent adjudication would establish whether discordant objects are genuine, whereas the paired comparison would estimate whether incremental detection is attributable to AI rather than preparation composition. We propose distinguishing “additional detection on a Techcyte-prepared slide,” “confirmed additional detection,” and “AI-attributable diagnostic gain.”

## Data Availability

No new data were generated or analyzed for this Comment Letter.
